# Structural, Interfacial, Gelling, and Digestive Properties of Protein from *Grifola frondosa* Fruiting Body

**DOI:** 10.3390/gels12050412

**Published:** 2026-05-09

**Authors:** Yu Wang, Shuyu Song, Qiuyan Liu, Lihong Chen, Weimin Liu, Juan Wu, Yu Cheng

**Affiliations:** 1 School of Food and Biological Engineering, Jiangsu University, 301 Xuefu Road, Zhenjiang 212013, China; 2222318068@stmail.ujs.edu.cn (Y.W.); 3230905007@stmail.ujs.edu.cn (S.S.); 2212318016@stmail.ujs.edu.cn (Q.L.); bush_hong@ujs.edu.cn (L.C.); liuwmwu@ujs.edu.cn (W.L.); 2 Institute of Food Physical Processing, Jiangsu University, Zhenjiang 212013, China

**Keywords:** *Grifola frondosa* protein, gelation, digestion, interfacial property

## Abstract

Culture medium formulation influences mushroom yield and composition, but its effect on the properties of edible fungal protein remains unclear. To explore the functional and nutritional properties of proteins from *Grifola frondosa* (GF) fruiting bodies, the study examined the structural, interfacial, gelling, and digestive properties of GF proteins grown in four culture media. The four GF proteins obtained were labeled GFP1–GFP4, respectively. The β-turn content and intrinsic fluorescence in GFP1 increased by 41.48% and 36.45% (*p* < 0.05), respectively, compared to GFP4. GFP4 exhibited higher surface pressure at the air–water interface and lower interfacial force at the oil–water interface. In comparison with GFP4, the other GFPs showed a higher rate of interfacial film formation and greater film elasticity and strength. GFP2 had a minimum gelling concentration of 80 mg/mL, which is a 33.33% reduction from GFP4. The storage modulus (G′) of GFP1 was 58 times higher than that of GFP4 (10 Pa), indicating a significant increase in gel elasticity (*p* < 0.05). Additionally, compared to GFP4, GFP1 showed a 16.59% increase in total amino acid and a 6.82% increase in free amino group release (*p* < 0.05), although its digestibility decreased by 5.06% (*p* < 0.05). These results suggest that the formulation of the culture medium alters the structures and interfacial properties of GFPs, thereby impacting their functionalities and applications in food colloid-based products.

## 1. Introduction

Animal protein has traditionally constituted a significant portion of the diet due to its complete amino acid profile and high digestibility. However, livestock farming requires large amounts of land, generates effluents that exacerbate freshwater eutrophication, and emits greenhouse gases, which are major contributors to climate change [1,2]. Research indicates that excessive consumption of red meats such as pork and beef can lead to obesity and chronic illnesses [3]. Recently, to lessen the environmental impact of animal protein sources, there has been increased interest in finding sustainable alternative proteins that can partially replace animal-based sources and reduce overall consumption [4,5,6].

In addition to plants, algae, and insects, fungi are currently being explored as alternative protein sources [7,8,9]. Edible fungi production primarily uses industrial and agricultural wastes as raw materials. This method offers benefits, including three-dimensional growth, reduced land use, and compliance with low-carbon and environmental protection goals. Consequently, producing edible fungi protein presents a new strategy to enhance food production systems and support the nitrogen cycle in ecosystems [10]. Protein from edible fungi boasts a rich amino acid profile [7], with essential amino acids making up 35–50% [8], high digestibility [11], low allergenicity [12], and consumer acceptance. It stands out as a promising alternative protein source.

*Grifola frondosa* is a medicinal and edible fungus known for its distinctive aroma and value [13,14]. Its fruiting bodies contain approximately 19.3% protein, slightly above the typical 17.2% found in other edible fungi [15]. Therefore, GFP can be used as a potential alternative protein source. Much research has explored the physiological functions of GFP [16,17,18] and their hydrolysates [19,20]. Nonetheless, few studies have investigated GFP’s digestive properties and its functionalities. Multiple freeze–thaw treatments have been shown to simultaneously enhance the emulsifying properties and digestibility of GFPs [21]. When GFP was crosslinked with transglutaminase and subjected to thermal treatment, its emulsifying properties and inhibition of fat digestion improved [22].

In addition to modifying GFP itself, medium formulations also play a key role in its functional properties. Currently, *Grifola frondosa* fruiting bodies are primarily cultivated on wood chips and cottonseed hulls. However, cottonseed hulls have low protein content and poor conversion efficiency. Research indicates that different cultivation substrates influence the growth and composition of *Grifola frondosa* [23,24]. In addition, substituting cottonseed hulls with high-protein agricultural by-products, such as rice bran, wheat bran, and corn flour, as carbon and nitrogen sources can enable sustainable, high-value utilization of these by-products [24,25] and might improve the properties of GFP [26].

To better understand how medium composition influences GFP properties, proteins from GF fruiting bodies grown in four culture media with varying proportions of cottonseed hulls, wheat bran, corn flour, and rice bran were obtained. Then, the structure, digestibility, interfacial, and gelling properties of these four GFPs (GFP1, GFP2, GFP3, GFP4) were assessed.

## 2. Results and Discussion

### 2.1. Structural Characterization of GFP

#### 2.1.1. Amino Acid Composition

The amino acid composition of four GFP samples is presented in Table 1. The EAA/TAA ratios for all four GFPs were approximately 41%. Compared with GFP4, the total amino acid contents of GFP1 and GFP2 increased by 16.59% and 18.16% (*p* < 0.05), respectively. All four GFPs exhibited high histidine content, with amino acid scores greater than 1, indicating that GFP can serve as a complementary protein source for histidine. Whey protein, a high-quality animal protein, is histidine-limited [27]. This suggests that GFP and whey protein have complementary amino acid profiles and can be combined to produce composite protein products with higher nutritional value.

#### 2.1.2. FTIR

**Figure 1:**
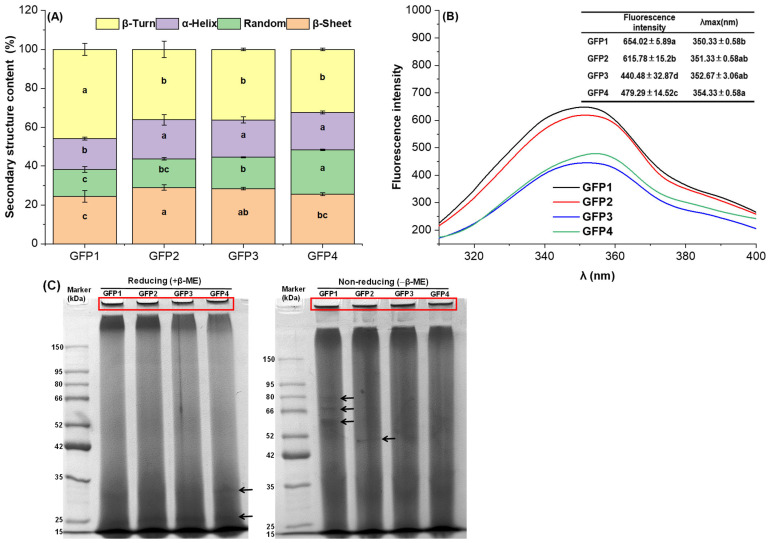
Secondary structure (**A**), fluorescence spectra (**B**), and SDS-PAGE (**C**) of the four GFP samples. Values in the same column sharing no common letters differ significantly (*p* < 0.05).

The amide I band absorption peak in the range of 1700–1600 cm^−1^ is primarily used to calculate protein secondary structure. The protein secondary structure of GFP samples is shown in Figure 1A. When compared with other samples, GFP1 exhibited the highest amount in β-turn content (*p* < 0.05), whereas GFP1 exhibited the lowest amount in α-helix, random coil, and β-sheet contents. The β-turn content of GFP1 was 41.48% higher than that of GFP4 (*p* < 0.05), while the α-helix and random coil contents were 16.74% and 38.89% lower than those in GFP4 (*p* < 0.05), respectively. GFP2 exhibited a higher β-sheet content, with a 11.70% higher β-sheet content than GFP4. These results indicate that the culture medium formulation affects the secondary structure of GFP. Our findings align with research on mung bean protein (MBP), suggesting that nitrogen fertilization can modify its secondary and tertiary structures [26].

#### 2.1.3. Fluorescence Spectroscopy

To further investigate the effect of culture medium formulation on the tertiary structure of GFP, the intrinsic fluorescence of tryptophan in GFP was shown in Figure 1B. The results indicated that, compared with GFP4, GFP1 and GFP2 showed significantly higher fluorescence intensities, with increases of 36.45% and 28.48%, respectively (*p* < 0.05), whereas GFP3 exhibited a significantly lower intensity, with a decrease of 8.10% (*p* < 0.05). This variation could be due to differences in the number of exposed tryptophan residues among GFP variants. Additionally, a blue shift in the maximum emission wavelength of GFP1 was observed relative to GFP4 (*p* < 0.05). This indicates that the media modifications altered the tryptophan microenvironment in GFP1, reducing its polarity and increasing its hydrophobicity [29,30,31]. Overall, these findings show that the composition of the culture medium impacts the tertiary structure of GFP.

#### 2.1.4. SDS-PAGE

The molecular weights of GFPs in the SDS-PAGE were shown in Figure 1C. Under reducing conditions, all four GFP samples showed a band exceeding 150 kDa, along with two smaller bands at approximately 25 and 35 kDa. Under non-reducing conditions, GFP1 showed three bands near 66 kDa, while GFP2 appeared as a single, distinct band at 42 kDa, corroborating previous findings [16,32]. A dark blue stain was evident in the loading wells of both the reduced and non-reduced gels for all GFP samples, suggesting the presence of large, aggregated proteins that did not migrate into the gel.

### 2.2. Digestive Properties of GFP

**Figure 2:**
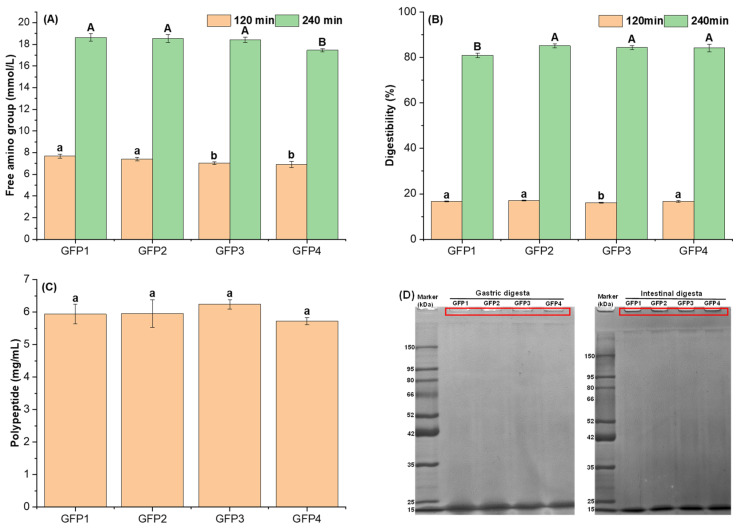
Free amino group content (**A**), digestibility (**B**), polypeptide content (**C**), and SDS-PAGE of gastric and intestinal digesta (**D**) for the GFPs. Different lowercase letters indicate significant differences at 120 min (*p* < 0.05); different uppercase letters indicate significant differences at 240 min (*p* < 0.05).

The digestibility of protein is essential for assessing its nutritional value, understanding how proteins are utilized, and guiding the development of related products. Figure 2 illustrates the digestive characteristics of the GFPs. The amount of free amino groups released from broken polypeptide bonds indicates the level of protein hydrolysis.

The free amino groups after the gastric digestion are shown in Figure 2A. Compared with the GPF4, the release of free amino group from the gastric digesta of GFP1 and GFP2 increased by 10.82% and 7.07%, respectively (*p* < 0.05), whereas GFP3 showed no significant difference (*p* > 0.05). This may be because GFP1 and GFP2 exhibited high Phe content (Table 2). Phe is one of the enzymatic sites of pepsin [33]. Moreover, compared with GFP4, the release of free amino groups in the intestinal digesta of GFP1, GFP2, and GFP3 increased by 6.82%, 6.19%, and 5.56%, respectively (*p* < 0.05). The digestibility after the intestinal digestion is shown in Figure 2B. The digestibility at the gastric stage was below 20% for all four GFP samples, whereas the digestibility in the intestinal stage exceeded 80%, indicating that GFP is primarily digested during the intestinal phase. Compared to GFP4, GFP1 and GFP2 showed no significant difference in digestibility at the gastric step, while that of GFP3 was reduced by 2.57% (*p* < 0.05). After the intestinal digestion, GFP2 and GFP3 exhibited no significant difference compared to GFP4, whereas the digestibility of GFP1 was 5.06% lower (*p* < 0.05).

Although digestion rates varied, there was no significant difference in polypeptide release during intestinal digestion among the groups (*p* > 0.05) (Figure 2C). Gel electrophoresis showed complete breakdown of high-molecular-weight proteins in GFPs (Figure 2D). Compared with the gel profiles in Figure 1C for the four GFP samples, the dark blue staining in the loading wells was absent, indicating that high-molecular-weight proteins had been broken down into smaller fragments. This indicates that GFPs did not differ in the ultimate protein digestibility but likely in their digestion kinetics.

### 2.3. Physicochemical Properties and Functionalities of GFP

#### 2.3.1. Zeta Potential

**Figure 3:**
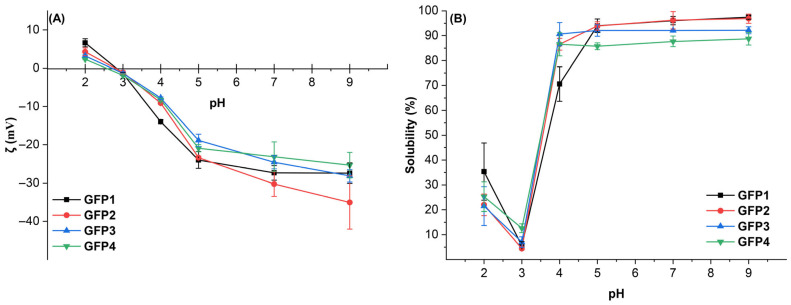
ζ-potential (**A**) and solubility (**B**) of the GFP samples.

As shown in Figure 3A, the ζ-potential of GFPs decreases as pH increases from 2.0 to 9.0. The ζ-potential of GFPs approached zero near pH 3.0, indicating that GFP’s isoelectric point is approximately at that pH. At pH 2.0, all four proteins carry a positive surface charge, although with relatively low potential. At pH 2.0, GFP1 exhibits the highest ζ-potential and shows 2.85 times higher ζ-potential than GFP4 (*p* < 0.05). Under mild acidic conditions (pH 4.0), GFP1’s absolute ζ-potential is 69.67% higher than that of GFP4 (*p* < 0.05). As pH increases from 4.0 to 7.0, the absolute ζ-potential of GFP1, GFP2, GFP3, and GFP4 increases by 95.64%, 233.11%, 215.55%, and 180.58%, respectively. From neutral pH 7.0 to alkaline pH 9.0, the increase in negative surface charge levels off, with GFP1, GFP2, GFP3, and GFP4 showing only 2.56%, 15.82%, 14.58%, and 0.95% increases in absolute ζ-potential, respectively. while at pH 9.0, GFP2 showed the highest absolute ζ-potential, which was 30.04%, 34.52%, and 36.59% higher than that of GFP1, GFP3, and GFP4, respectively. The net charge of GFPs at pH 9.0 was higher than that at pH 2.0. The reason may be the higher content of basic amino acids relative to acidic amino acids.

The change in ζ-potential indicates the electrostatic stability of colloidal particles. As pH moves away from the isoelectric point, the absolute ζ-potential increases and electrostatic repulsion increases, leading to partial protein unfolding. This may influence protein solubility and dispersion stability [34,35]. The high fluorescence intensities of GFP1 and GFP2 confirmed partial unfolding or extension of their tertiary structures (Figure 1B). Overall, GFP1 and GFP2 displayed slightly higher absolute ζ-potential values compared to GFP3 and GFP4. Consequently, GFP1 and GFP2 are likely to exhibit higher solubility.

#### 2.3.2. Solubility

Solubility is a crucial indicator for assessing protein applications in food processing because it affects functionalities such as foaming and emulsification [35]. The solubility of the four GFP samples varied with pH, as shown in Figure 3B. When the pH decreased from 3.0 to 2.0, the solubility of GFP1, GFP2, GFP3, and GFP4 increased to 6.33-fold, 4.92-fold, 3.06-fold, and 2.01-fold, respectively (*p* < 0.05). However, there was little difference in solubility among the four GFPs (*p* > 0.05). In contrast to the moderate increase observed with acidification, alkalization greatly enhanced GFP solubility. As pH rose from 3.0 to 4.0, the solubilities of GFP1, GFP2, GFP3, and GFP4 increased by 12.63-, 19.33-, 12.89-, and 6.86-fold, respectively (*p* < 0.05). It was not surprising because charged amino acid residues can enhance protein-water interactions, thereby improving protein solubility [36]. While the net charge at pH 4.0 was higher than that at pH 3.0, as indicated by the zeta-potential. Additionally, GFP2 and GFP3 showed little difference in solubility from GFP4, whereas GFP1’s solubility was notably lower by 18.44% (*p* < 0.05) than that of GFP4.

The solubility of GFP2, GFP3, and GFP4 remained constant from pH 4.0 to pH 9.0, while GFP1’s solubility did not change from pH 5.0 to pH 9.0. At pH 7.0, the solubility of GFP1, GFP2, and GFP3 was higher than GFP4 by 9.50%, 9.69%, and 4.94%, respectively (*p* < 0.05). It’s not surprising that solubility increases when the pH deviates from the pI. When pH deviates from the isoelectric point, either increasing or decreasing pH can raise the net surface charge of the protein. This is supported by the GFP ζ-potential data shown in Figure 3A. A higher potential improves interactions between the protein and water, thereby boosting solubility. Our findings align with other studies showing that solubility differences are linked to variations in surface charge [37]. Protein beverages and juice blends typically have acidic or neutral pH, making GFP’s high solubility under these conditions advantageous for food applications such as beverages [34].

#### 2.3.3. Foaming Property

**Figure 4:**
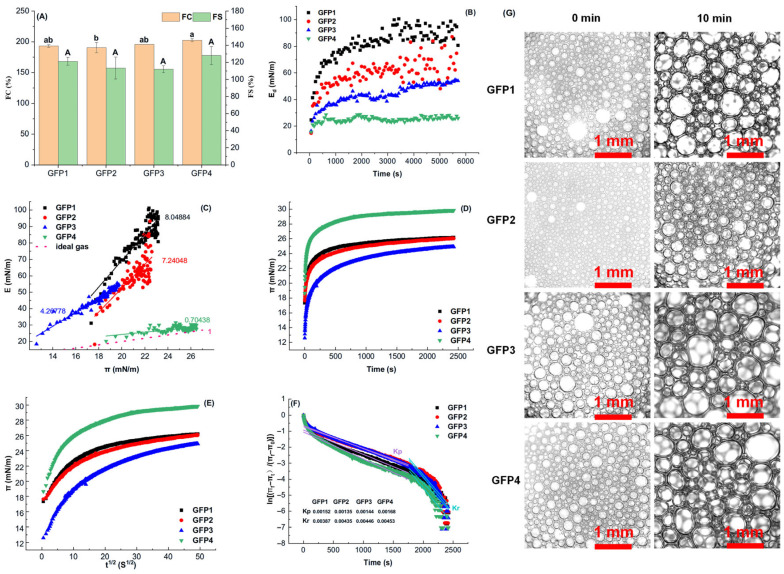
Foaming properties (**A**), interfacial properties at the air–water interface (**B**–**F**), and foam microstructure (**G**) of the GFPs. ((**B**) Dynamic dilatational elastic modulus (Ed) versus time (t) curves for the GFPs at the air–water interface; (**C**) Dilatational elastic modulus (**E**) versus surface pressure (π) curves at the air–water interface; (**D**,**E**) Time-dependent dynamic surface pressure profiles, presented as π–t and π–t^1/2^ curves, respectively; (**F**) Typical profile depicting the penetration and rearrangement steps of protein molecules at the interface. Kp and Kr represent the first-order rate constants for penetration and rearrangement, respectively.) Different lowercase letters indicate significant differences in FC (*p* < 0.05); different uppercase letters indicate significant differences in FS (*p* < 0.05).

Foams, owing to their ability to provide unique structures, appearances, and mouthfeel, serve as crucial components in certain foods, such as carbonated beverages, ice cream, mousse, and whipped cream [38]. To evaluate the foaming properties of GFPs, the FC and FS of GFPs at pH 7.0 were investigated, as shown in Figure 4A. Compared to GFP4, the FC of GFP1 and GFP2 showed no significant increase. Notably, the FC of GFP3 was 6.30% lower than that of GFP4 (*p* < 0.05). No significant differences were observed in the FS among the GFPs (*p* > 0.05).

Microscopy analysis of the foam microstructure, shown in Figure 4G, revealed that all four GFP samples had uniformly dispersed spherical bubbles after whipping. This is likely due to their high solubility, which enhances protein dispersion at the air–water interface and promotes the formation of dense, consistent bubbles. After 10 min, gravity-driven drainage and Ostwald ripening, in which smaller bubbles dissolve and merge into larger ones, led to bubble growth. While they retained their spherical shape, they became more polygonal, with small bubbles surrounding larger ones, resulting in a sparse appearance. These results align with previous studies [3,39]. Overall, these results indicate minimal differences in FS among the GFPs, consistent with the data in Figure 4A.

Since the foaming properties of proteins are closely linked to air–water interface stability [40,41], the surface tension and surface viscoelasticity at this interface were analyzed to better understand the foaming performance of the GFPs. The surface pressure (π) evolution at the air–water interface is shown in Figure 4D. The initial rapid increase and subsequent plateau indicate GFP adsorption and a reduction in interfacial tension. Saturation was reached after approximately 500 s. The interfacial pressure-lowering ability followed the order GFP4 > GFP1 ≥ GFP2 > GFP3, consistent with the FC trend in Figure 4A. The π–t^1/2^ plots (Figure 4E) revealed significant deviation from linearity, suggesting that adsorption involves not only diffusion but also protein unfolding, penetration, and rearrangement [42]. Penetration (K_p_) and rearrangement (K_r_) rate constants, derived from a first-order model (Figure 4F), generally followed the same trend as the π values.

The dilatational elastic modulus is useful for examining intermolecular interactions and protein structure during adsorption at the interface [41,43]. The E_d_ curves for GFP solutions at the air–water interface are shown in Figure 4B. In all samples, the elastic modulus significantly exceeded the viscous modulus, approaching the E value. This suggests that the protein films on GFP-stabilized droplets are primarily elastic-viscoelastic. As the adsorption time increased, the E_d_ value gradually increased, suggesting protein unfolding at the interface. This strengthened intermolecular interactions and increased protein adsorption [41]. During the time-scan stage, E_d_ values rose gradually from GFP4 to GFP1. At the steady stage, GFP1’s interfacial film exhibited the highest viscoelasticity, with E_d_ increasing by 70.22% relative to GFP4.

The π and elastic modulus of protein adsorption films at the oil–water interface are affected by processes such as protein adsorption, unfolding, folding, and surface coverage. Consequently, the change in the dilatational modulus (E) with π was examined to provide a useful method for analyzing non-ideal interfacial behavior in adsorption. The curves of the E for films formed by GFP solutions from different cultivation media at the air–water interface are shown in Figure 4B. Additionally, the slopes of the E–π plots during the adsorption phase are displayed in Figure 4C.

The E values for GFPs rose with increasing π values, especially for GFP1, GFP2, and GFP3, although not in a perfectly linear way. This pattern indicates that the E value depends on the amount of protein adsorbed and is affected by intermolecular interactions, demonstrating the presence and persistence of forces among the adsorbed protein residues on the film surface. Additionally, at the air–water interface, the slope of the E–π curve increased notably from GFP4 to GFP1. GFP4 had the smallest E–π slope, about 91.25% lower than GFP1’s. The slopes for GFP1 (8.049), GFP2 (7.240), and GFP3 (4.268) were well above 1 (as indicated by the dashed line in Figure 4C), indicating non-ideal behavior and strong interactions among the interfacially adsorbed proteins. Conversely, GFP4’s slope (0.704) was below 1, implying weaker protein–protein interactions at the interface [44]. Furthermore, from GFP1 to GFP4, both the elastic modulus and the amount of protein adsorption at the interface gradually decreased. These findings agree with those shown in Figure 4B.

#### 2.3.4. Emulsification Property

**Figure 5:**
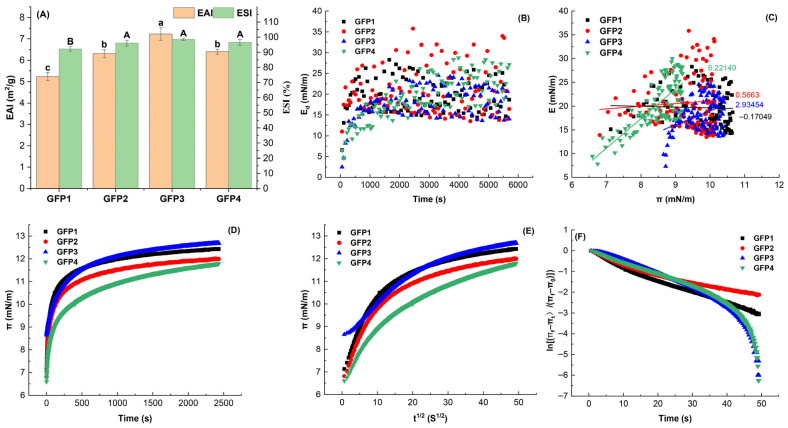
Emulsifying properties (**A**) and oil–water interfacial properties (**B**–**F**) of the GFPs. (Note: (**B**) Dynamic dilatational elastic modulus (Ed) versus time (t) curves at the oil–water interface; (**C**) Dilatational elastic modulus (**E**) versus surface pressure (π) curves at the oil–water interface; (**D**) and (**E**) Time-dependent dynamic surface pressure profiles, presented as π–t and π–t^1/2^ curves, respectively; (**F**) Typical profile depicting the penetration and rearrangement steps of protein molecules at the interface). Different lowercase letters indicate significant differences in EAI (*p* < 0.05); different uppercase letters indicate significant differences in ESI (*p* < 0.05).

As depicted in Figure 5A, GFP2’s EAI showed no significant difference from GFP4 (*p* > 0.05), while GFP1’s was reduced by 18.26%, and GFP3’s increased by 12.79% (*p* < 0.05). Proteins adsorb at interfaces and decrease π, thereby promoting rapid droplet formation. A higher EAI indicates faster protein adsorption to oil droplet surfaces, thereby facilitating the formation of the oil–water interface. Thus, the oil–water interfacial characteristics of proteins serve as indicators of their emulsifying ability. To further explore the emulsifying properties of the GFPs, their surface tension and surface viscoelasticity at the oil–water interface were measured.

The π of the GFP solutions initially rose quickly and then leveled off over time, as illustrated in Figure 5D. This suggests that GFP molecules adsorb at the oil–water interface, decreasing the interfacial π. Adsorption reached a saturation point after about 1000 s for each sample, after which the π stayed constant. According to the figure, the effectiveness of the GFP samples in reducing oil–water interfacial pressure followed the order GFP3 ≥ GFP1 > GFP2 > GFP4.

To study protein diffusion kinetics at the interface, the change in dynamic surface pressure (π–t^1/2^) curves for GFPs adsorbed at the oil–water interface is presented in Figure 5E. The π–t^1/2^ curves of the GFP solutions significantly deviate from a straight line, similar to those observed at the air–water interface in Figure 4E. This indicates that the adsorption process at the oil–water interface is mainly driven by unfolding, penetration, and reorganization of the protein structure at the interface [41,42,43].

The penetration and rearrangement rates of GFPs at the oil–water interface are shown in Figure 5F. The plot of ln [(π_f_ − π_t_)/(π_f_ − π_0_)] versus time (t) reveals two linear regions. Notably, GFP1 and GFP2 do not exhibit a clear linear region associated with rearrangement, possibly because they require longer times to penetrate and rearrange. Conversely, GFP3 and GFP4 exhibited similar trends.

The ESI of GFP2 and GFP3 was comparable to GFP4 (*p* > 0.05), whereas GFP1 showed a 4.43% decrease (*p* < 0.05). A higher ESI suggests a greater protein capacity to prevent phase separation in emulsions [31,45]. The curves depicting the E_d_ of the adsorbed films formed by various GFPs at the oil–water interface are shown in Figure 5B. For all samples, the elastic modulus was notably higher than the viscous modulus and closely approached the E value, indicating that the protein adsorption films on GFP-stabilized droplets were mainly elastic viscoelastic films. As the adsorption time increased, the dynamic E_d_ gradually increased, indicating that proteins at the oil–water interface unfolded, thereby strengthening intermolecular interactions and enhancing protein adsorption [31,41,43]. In Figure 5B, during the early moments of the time-scan stage, E_d_ values rapidly increased for all GFP samples except GFP4, which exhibited a slower rise. Ultimately, the final E_d_ values of GFPs showed no significant differences.

Similarly, the variation in the E with π was used to examine nonideal interfacial behavior during adsorption. The E curves for the GFP solutions from different cultivation media at the oil–water interface, along with the slopes of the E–π plots during adsorption, are shown in Figure 5C. As observed at the air–water interface (Figure 4C), E values for GFPs at the oil–water interface increased with rising π, indicating the presence and stability of intermolecular forces among adsorbed protein residues within the film. Additionally, the slopes of the E–π plots for GFP3 (2.934) and GFP4 (6.221) were significantly greater than 1 (represented by the dashed line in Figure 5C), suggesting nonideal behavior and considerable interaction among interfacial adsorbed protein molecules. Conversely, the slopes for GFP1 (−0.170) and GFP2 (0.566) were less than 1, indicating weaker protein–protein interactions at the interface [44]. This pattern aligns with the EAI results shown in Figure 5A.

#### 2.3.5. Rheological Properties and Minimum Gelling Concentration (MGC)

**Figure 6:**
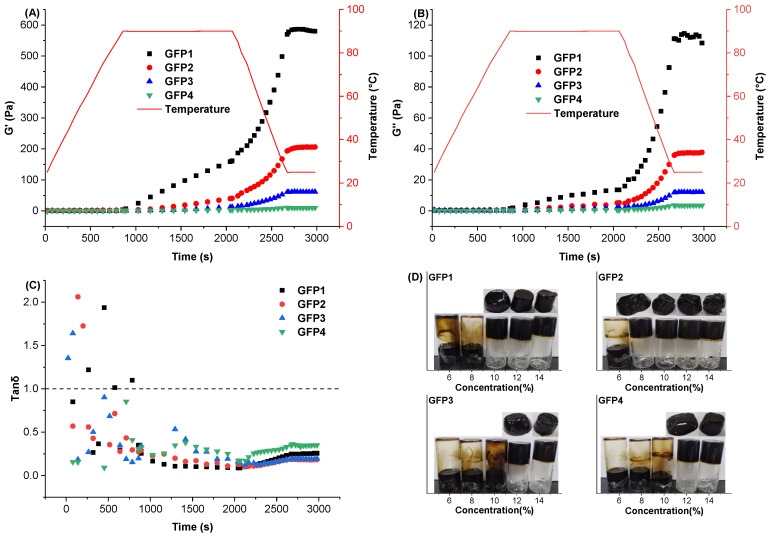
Storage modulus (**A**), loss modulus (**B**), loss tangent (**C**) during gelation, and MGC (**D**) of the GFPs.

Temperature scanning reveals the structure of heat-induced gelation in protein aggregates. By monitoring changes in storage modulus (G′), loss modulus (G″), and loss tangent (tanδ = G″/G′) with temperature for GFP at 100 mg/mL, gel formation behavior was observed. The results are presented in Figure 6A–C. At each temperature, G′ was greater than G″, and tan δ was less than 1, indicating a gel phase with elastic rather than viscous properties. Notably, GFP4 exhibited approximately 10 Pa at 0.5% strain, indicating very weak gelation. During the temperature scan, the G′ and G″ values of GFP1, GFP2, and GFP3 were higher than those of GFP4. The final G′ values increased by 57.50-fold, 19.50-fold, and 5.10-fold, respectively, while G″ increased by 31.46-fold, 9.71-fold, and 2.26-fold, respectively (*p* < 0.05). GFP1 exhibited the highest G′, followed by GFP2, reflecting the gel state of these samples at their minimum gelling concentration, as shown in Figure 6.

The MGC and gel state of GFPs are shown in Figure 6D. The MGCs of GFP1, GFP2, GFP3, and GFP4 were 100, 80, 120, and 120 mg/mL, respectively. Compared with GFP4, GFP1 and GFP2 showed reductions of 16.67% and 33.33% in MGC, respectively, with GFP2 having the lowest concentration. This is likely because it had the highest β-sheet content (Figure 1A), which promotes gelation [46,47,48].

**Figure 7:**
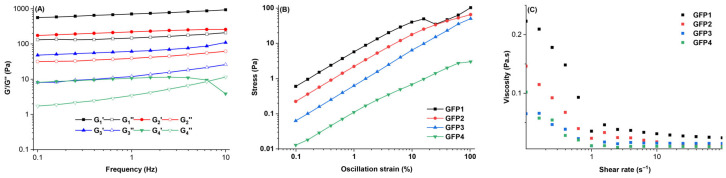
Frequency sweep (**A**) and amplitude sweep (**B**) of the GFPs gels, and apparent viscosity of the GFPs solutions (**C**).

Frequency-sweep analysis was employed to assess the viscoelastic behavior and response of protein gels over a range of frequencies. As shown in Figure 7A, both G′ and G″ of all GFP gels increased as shear frequency rose, with G′ consistently higher than G″. This suggests the presence of a continuous, network-like structure within the GFP gels [26]. These indicated that GFP gels were supportable and exhibited solid-like properties [49].

Amplitude sweep reflects the mechanical properties of gels relevant to food processing and also reveals the diversity of gel structure. Fracture stress normally reflects gel hardness, whereas shear strain reveals gel brittleness [50]. As shown in Figure 7B, the shear stress of GFPs increased with increasing strain force. The gels’ deformation increased until it reached the breaking point (the peak stress on the curve), after which it declined. Compared with GFP4, the shear stress of the other gel samples increased, indicating enhanced brittleness, while their fracture stress increased by 10–50 times.

Apparent viscosity describes how solutions flow and partly governs fluid movement in food-processing pipelines. The apparent viscosity of protein suspensions primarily depends on the free energy required to create space for molecular or aggregate motion. Figure 7C displays the apparent viscosity of GFP from various cultivation media. All GFP solutions showed shear-thinning behavior at low shear rates [51], typical of non-Newtonian fluids. As the shear rate increased, molecular chains likely aligned with the shear direction, decreasing apparent viscosity until it reached a steady level. The Herschel–Bulkley model is widely used to describe the flow behavior of non-Newtonian food fluids. It combines the Bingham plastic and power-law models and provides three parameters to describe the relationship between shear rate and shear stress [52,53,54]. The yield stress and shear rate of the emulsions fit the Herschel–Bulkley equation, and the parameters are shown in Table 3. All GFP samples had *n* values between 0 and 1, confirming strong shear-thinning behavior. The shear-thinning nature of GFP, with apparent viscosity decreasing and then stabilizing, suggests that it can be readily ingested and dispersed during food processing.

### 2.4. Principal Component Analysis (PCA)

**Figure 8:**
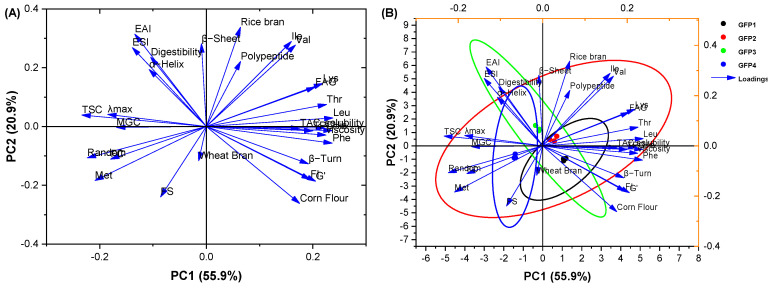
Principal component analysis (PCA) loading plot (**A**) and biplot (**B**) of the GFPs.

PCA was performed to examine how medium components influence GFP protein characteristics (Figure 8). Two components (PC1 + PC2) explained 76.80% of the total variance, with PC1 contributing 55.90% and PC2 20.90%. The loading plot (Figure 8A) shows that in PC1, rice bran and corn flour contents are positively loaded, whereas wheat bran is negative. Total protein content correlates positively with rice bran/corn flour but negatively with wheat bran. Solubility, apparent viscosity, and certain amino acids (Leu, Thr, Phe) were strongly associated with total protein. Free amino group release correlated with Lys content, and the elastic modulus links to fluorescence intensity and β-turn content. In PC2, digestibility is positively correlated with EAI, ESI, and α-helix content, while MGC associates with total sugar, random coil, and fluorescence emission wavelength. Collectively, PCA demonstrates that media rich in rice bran and corn flour (GFP1) enhance total protein content, β-turn structure, fluorescence intensity, and gel elasticity, whereas higher wheat bran content (GFP4) is associated with lower protein levels and distinct interfacial behavior.

The biplot can be used to combine the score and loading plots, revealing grouping by distance [55]. It reveals relationships between variables and between clusters [55,56,57]. Figure 8B shows a clear separation between the groups. GFP1 lies in the positive PC1/PC2 quadrant with the smallest ellipse, indicating high stability. GFP2 is also in the positive quadrants but near zero, with the largest ellipse, suggesting lower stability. GFP3’s tilted ellipse indicates a negative correlation between PC1 and PC2 within this group. These results demonstrate that medium composition alters GFP’s composition, structure, and functional properties. The clear separation of the four GFPs in the biplot confirms that medium composition systematically modulates the structural, interfacial, and gelling properties of GFP.

## 3. Conclusions

The results demonstrated that altering the cultivation medium produced significant differences in the characteristics of the GFP protein. Variations in the formulation of the cultivation medium increased the essential amino acid content and changed the secondary and tertiary structures of GFPs. As a result, properties such as zeta potential, solubility, foaming and emulsifying abilities, interfacial characteristics, and MGC of GFPs were altered. GFP1, GFP2, and GFP3 indicated higher interfacial film formation rates and greater interfacial film elasticity and strength. Additionally, GFP obtained from cultures grown in different media differed in apparent viscosity and gel elasticity, reflecting variations in rheological behavior. GFP1 and GFP2 exhibited higher gel elasticity. From the perspective of comprehensive functionality (gel elasticity, amino acid composition, and digestibility) and food-gel applications, GFP1 is considered the most promising candidate in practice. Moreover, differences in the medium also affected digestibility and the release of free amino groups during digestion. PCA indicated that total protein content in GFP correlated positively with rice bran and corn flour levels. Incorporating appropriate proportions of rice bran, wheat bran, and corn flour into the cultivation formulation beneficially modifies GFP properties.

Further research should examine how processing conditions (e.g., protein concentration, pH, ionic strength, physical treatments) affect the functionality of GFPs in industrial applications. While this study links medium formulation to GFP structure, the mechanisms underlying this relationship remain unclear. Future work could integrate omics to explore regulatory mechanisms.

## 4. Materials and Methods

### 4.1. Materials

The *Grifola frondosa* (GF) strain “Qinghui 152” (supplied by the Laboratory of Qingyuan Edible Fungi Research Institute (Lishui, China)) was used for cultivation and production of fruiting bodies. Medium ingredients, including Rice bran, wheat bran, corn flour, wood chips, cottonseed hulls, fine soil, brown sugar, and gypsum powder, were purchased from multiple local stores in Qingyuan, China. All chemical reagents were supplied by Shanghai National Pharmaceutical Group (Shanghai, China).

### 4.2. Cultivation of Grifola frondosa (GF)

GF was cultivated in four media (Table 2) that differed in the amounts of rice bran, wheat bran, corn flour, and cottonseed hulls. All ingredients were sequentially added to a substrate mixer and then combined with an equal weight of deionized water (160 kg). After blending, the media were divided into 200 substrate bags (1.6 kg each), placed in an autoclave, and sterilized at 121 °C for 12 h, then cooled to room temperature in a laminar flow cabinet. Holes (3 cm in diameter) were punched at each end of each bag, and the openings were quickly sealed by inserting two conical plugs cut from the “Qinghui 152” solid spawn. The inoculated bag was then covered with a transparent plastic bag and placed in a cool, dark location at 23 °C. It was maintained for approximately 2 months until dense white mycelium fully covered the substrate surface. The substrate was then transferred to a greenhouse and placed on fine, soft red soil, with the inoculated side facing up. It was cultivated for approximately 1 month at 23 °C and 65% relative humidity before harvesting.

### 4.3. Extraction of GFP

The procedure of Li et al. (2025) was used with slight modifications [22]. Fresh GF was dried at 40 °C for 2 days, then ground into a powder with a high-speed grinder. The powder was sieved through a 60-mesh screen and mixed with 95% ethanol at a 1:4 (g/mL) ratio. The mixture was stirred at 40 °C for 2 h to remove fats, then centrifuged at 10,000 *g* for 15 min to collect the precipitate. This step was repeated twice. The final precipitate was dried in a fume hood to produce defatted GF powder.

The defatted GF powder was combined with distilled water at a 1:40 (g/mL) ratio. After thorough mixing, the suspension pH was adjusted to 10.0. It was then stirred at 40 °C for 2 h to promote extraction, followed by centrifugation at 10,000 *g* for 15 min to obtain the supernatant. The supernatant was acidified to pH 3.0 using 2 mol/L HCl, then allowed to stand for 1 h, after which it was centrifuged at 10,000 *g* for 15 min. The resulting precipitate was collected and redissolved at a 1:10 (g/mL) ratio. This process was repeated once. The final precipitate was dissolved in an appropriate volume of distilled water, neutralized with 1 mol/L NaOH, and freeze-dried to yield four crude GFP samples (GFP1, GFP2, GFP3, and GFP4). GFP1, GFP2, GFP3, and GFP4 correspond to groups 1, 2, 3, and 4 in Table 2, respectively. The specific freeze-drying conditions for the sample were as follows: frozen at −20 °C for 12 h, then freeze-dried using an FD-8 freeze dryer (Beijing Persee General Instrument Co., Ltd., Beijing, China) at a pressure of 0–10 Pa for 72 h. The crude protein content in GFP1, GFP2, GFP3, and GFP4 was 73.02%, 69.95%, 63.01%, and 57.42%, respectively, as determined by the Kjeldahl method with a factor of 6.25.

### 4.4. Structural Characterization of GFP

#### 4.4.1. Amino Acid Composition

The amino acid composition of the samples was determined using the method described by Wang et al. (2023) [58]. Amino acid analysis was performed by chromatography using the S433D amino acid analyzer (Sykam GmbH, Eresing, Germany). The essential amino acids ratio score (EAA) and amino acid score (AAS) were obtained as follows:

(1) $$EAA\left(\%\right)=\frac{EAA\;of\;sample\;(g/100\;g\;protein)}{EAA\;of\;egg\;protein\;(g/100\;g\;protein)}\times 100$$

(2) $$AAS\left(\%\right)=\frac{AA\;of\;sample\;(g/100\;g\;protein)}{AA\;of\;egg\;protein\;(g/100\;g\;protein)}\times 100$$

#### 4.4.2. Fourier Transform Infrared Spectroscopy (FTIR)

GFP and KBr were dried separately to remove moisture, then mixed at a ratio of 1:100–1:200 (*w*/*w*) and compressed into pellets. FTIR analysis of GFP was performed as described by Rehman et al. (2025) [59] in the range from 4000 to 400 cm^−1^ at a resolution of 4 cm^−1^ and 32 times scan. The spectral data were analyzed using Omnic 9.2 (Thermo Fisher Scientific, Waltham, MA, USA) and PeakFit 4.12 (Systat Software, Richmond, CA, USA). The relative percentages of each secondary structure were calculated by performing second derivative analysis or deconvolution fitting of the peak positions and sub-peak areas of the amide I band (1700–1600 cm^−1^).

#### 4.4.3. Intrinsic Fluorescence

The procedure of Yang et al. (2026) was followed with slight modifications [31]. GFP was dissolved in a phosphate buffer (10 mmol/L, pH 7.0) at a protein concentration of 0.2 mg/mL. The intrinsic fluorescence of GFP was measured with a F98 fluorescence spectrometer (Shanghai Lengguang Technology Co., Ltd., Shanghai, China) at an excitation wavelength of 295 nm and an emission wavelength of 300–450 nm, with a slit width of 10 nm.

#### 4.4.4. Sodium Dodecyl Sulfate-Polyacrylamide Gel Electrophoresis (SDS-PAGE)

GFP was dissolved at a protein concentration of 10 mg/mL in a reducing sample buffer containing 200 mmol/L β-mercaptoethanol. The samples were boiled for 5 min and then centrifuged at 10,000 *g* for 10 min. The GFP composition was analyzed by SDS-PAGE with a 5% stacking gel and a 12% separation gel, following the method described by Cheng et al. (2024) [29]. The electrophoresis voltage was initially set to 80 V and increased to 120 V as the samples moved through the separation gel. Gels were stained with Coomassie Brilliant Blue R-250 (Shanghai Macklin Biochemical Technology Co., Ltd. (Shanghai, China) for 60 min, then decolorized with a solution of methanol (10%) and acetic acid (10%) until the bands became clearly visible.

### 4.5. Digestive Properties of GFP

The digestion was conducted according to the procedure of Li et al. (2025) [22] with slight modifications, using an in vitro static gastrointestinal digestion model described by Brodkorb et al. (2019) [60]. Fifteen milliliters of GFP solution (protein content of 10.0 mg/mL) were mixed with 12 mL of simulated gastric fluid and 2.8 mL of water, then pH-adjusted to 3.0 with 0.2 mL of 1 mol/L HCl. Pepsin (2000 U/mL) and 7.5 μL of 0.3 mol/L CaCl_2_ were added, and the mixture was incubated at 37 °C with stirring at 100 r/min for 2 h. After adjusting the pH to 7.0 using 2 mol/L NaOH, the gastric digesta was combined with 16 mL of simulated intestinal fluid (SIF), and the total volume was brought to 40 mL with ultrapure water. The pH was maintained at 7.0 with 0.1 mL of 2 mol/L NaOH, and 40 µL of 0.3 M CaCl_2_ was added. Porcine bile salts (final concentration of 10 mmol/L) and pancreatin (final concentration of 100 U/mL trypsin) were then added. The intestinal digestion was performed at 37 °C with stirring at 100 rpm for 2 h. From this, 0.5 mL samples of gastric and intestinal digesta were collected and immediately heated at 90 °C for 15 min. Protein degradation was analyzed via SDS-PAGE as described in Section 4.4.4. Polypeptide content was measured using the biuret method after removing intact proteins with 20% TCA. The free amino groups in the supernatants were determined by the OPA method [61]. Digestibility was calculated accordingly:

(3) $$Digestibility\left(\%\right)=\frac{C_{t}}{C_{0}}$$

where C_t_ is the free amino acid group content at the endpoint digesta, and C_0_ is the total free amino acid group content of the sample before digestion.

### 4.6. Functionalities of GFP

#### 4.6.1. Solubility

GFP dispersion was prepared by dissolving 25.0 mg of GFP powder in 100 mL of distilled water, then using 2 mol/L NaOH and 1 mol/L HCl to adjust the pH to 2.0, 3.0, 4.0, 5.0, 7.0, and 9.0. The GFP dispersion was centrifuged at 10,000 *g* for 10 min, and the supernatant was collected. The protein concentration in the supernatant was determined using the Lowry method [62]. The solubility was obtained according to the following formula:

(4) $$Solubility\left(\%\right)=\frac{protein\;content\;in\;the\;supernatant}{protein\;content\;in\;GFP\;powder}\times 100$$

#### 4.6.2. Foaming Property

The foaming properties were determined using the method described by Chen et al. (2025) [63]. The GFP solution was prepared in 10 mmol/L phosphate buffer (pH 7.0) with a protein concentration of 10 mg/mL. A 10 mL sample of GFP solution was transferred into a 50 mL measuring cylinder and homogenized at 10,000 r/min for 3 min. The foam volume was recorded immediately, and the foam was allowed to stand for 10 min before measuring the residual volume. Foam capacity (FC) and foam stability (FS) were then calculated with the following formulas:

(5) $$FC\left(\%\right)=\frac{V_{0}}{V}\times 100$$

(6) $$FS\left(\%\right)=\frac{V_{10}}{V}\times 100$$

where V is the volume of the initial protein solution (mL), V_0_ is the foam volume at 0 min after shearing (mL), and V_10_ is the foam volume after standing for 10 min (mL).

#### 4.6.3. Microscopic Image of Foam

Foam formed at 0 and 10 min after shearing was sampled with a pipette onto a concave glass slide. The foam microstructure was observed with a 4× objective lens on a microscope (BM2000, Jiangnan Yongxin Optical Co., Ltd., Nanjing, China), and the images were recorded accordingly.

#### 4.6.4. Emulsifying Property

The GFP solution was prepared in 10 mmol/L phosphate buffer (pH 7.0) at a protein concentration of 10 mg/mL. A 12 mL aliquot of GFP solution and 1.2 mL of corn oil were homogenized at 10,000 rpm for 3 min in a 50 mL centrifuge tube using a high-speed homogenizer. The absorbance at 500 nm was measured using a VIS-7220N spectrophotometer (Shanghai Xinmao Instrument Co., Ltd., Shanghai, China). The emulsifying activity index (EAI) and emulsion stability index (ESI) of the resulting emulsion were assessed to indicate GFP’s emulsifying properties, following the method described by Shen et al. (2025) [45]. EAI and ESI were calculated using the following equations:

(7) EAIm2g=2×2.303×N×A0C×φ×104

(8) $$ESI\left(\%\right)=\frac{A_{30}}{A_{0}}\times 100$$

where N indicates the dilution factor; A_0_ and A_30_ indicate the absorbances of the diluted emulsion at 500 nm at 0 and 30 min; C indicates the protein concentration, g/mL; and φ indicates the volume fraction of the aqueous phase in the emulsion.

#### 4.6.5. Minimum Gelling Concentration (MGC)

Four grams of GFP solution at protein concentrations of 60, 80, 100, 120, and 140 mg/mL were transferred to flat-bottomed tubes, sealed with aluminum foil, and heated at 90 °C for 30 min. After heating, the samples were rapidly cooled to room temperature and stored at 4 °C overnight. The next day, they were equilibrated at room temperature for 1 h, then gently inverted to indicate the MGC. A sample was considered to have formed a gel if it did not move when the inverted tube was rotated [39].

#### 4.6.6. Rheological Properties

A 100 mg/mL GFP solution (10 mM PB, pH 7.0) was prepared for rheological analysis. Temperature-cycling tests were performed using a Discovery HR-1 hybrid rheometer (TA Instruments, New Castle, DE, USA) according to the methods reported by Liu et al. (2026) [64] with a parallel-plate geometry (diameter = 40 mm, gap = 1000 μm). GFPs were heated from 25 °C to 90 °C at a rate of 5 °C per minute, maintained at 90 °C for 30 min, then cooled back to 25 °C at 5 °C per minute. The temperature sweep was conducted at 1 Hz and at a strain of 1% for GFP1, GFP2, and GFP3, and 0.5% for GFP4. Frequency- and strain-sweep measurements were performed on the gels at 25 °C in oscillatory mode after the temperature cycle. The applied frequency ranged from 0.1 to 10 Hz at a strain of 1%. The applied strain ranged from 0.1% to 100% at a frequency of 1 Hz.

The apparent viscosity and yield stress were determined at the shear rate of 0.1–100 s^−1^, as described by Li et al. (2025) [22]. The relationship between yield stress and shear rate was fitted to the Herschel–Bulkley model [52,54]. The model formula is as follows:

(9) $$\sigma =\sigma_{0}+K{\overset{\cdot }{\gamma }}^{n}$$

where σ represents the shear stress (Pa), σ_0_ represents the yield stress, $\overset{\cdot }{\gamma }$ denotes shear rate (s ^−1^), K represents the apparent viscosity coefficient, and n indicates the flow index.

### 4.7. Physicochemical Properties of GFP

#### 4.7.1. Ζeta Potential

The zeta potential of the GFP solutions (1 mg/mL) at different pH values (2.0–9.0) was measured using Anton Paar Litesizer 500 (Anton Paar, Graz, Austria) at 25 °C, with an equilibration time of 1 min.

#### 4.7.2. Interfacial Rheology

The GFP solution was prepared in a 10 mmol/L phosphate buffer (pH 7.0) with a protein concentration of 5 mg/mL. The interfacial pressures at the air-liquid and oil–water interfaces were measured following the method outlined by Kontogiorgos et al. (2023) [41]. Measurements were conducted at 25 °C using a Krüss DSA 100 s drop shape analyzer (Krüss, Hamburg, Germany), with a single measurement comprising 10,000 repetitions at 1 s intervals. The experiment was manually stopped after 2400 s. The interfacial pressure was calculated using the following formula:

π = σ_0_ − σ_t_ (10)

where σ_0_ is the surface tension of the PB (10 mM, pH 7.0) at air-liquid (71.68 mN/m) and oil–water interfaces (23.34 mN/m), and σₜ represents the interfacial tension of the protein solution at time t.

The interfacial dilatational behavior of GFPs was measured at 0.1 Hz with a 10% amplitude for 5700 s. The protein’s diffusion rate at the air-liquid and oil–water interfaces was analyzed using the Ward-Tordai diffusion model, given by the following equation [65]:

π = 2C_0_KT(Dt/3.14)^1/2^ (11)

where C_0_ is the concentration of the sample, and K, T, t, and D represent the Boltzmann constant, absolute temperature, adsorption time, and diffusion coefficient, respectively.

A first-order equation was used to demonstrate the rate of protein penetration and rearrangement within the interfacial layer, as follows:

ln [(π_f_ − π_t_)/(π_f_ − π_0_)] = −k_i_t (12)

where π_f_, π_t_, and π_0_ represent interfacial pressure at the final adsorption time, the time t, and the initial time, respectively. k represents the first-order rate constant.

### 4.8. Statistical Analysis

All experimental procedures were conducted in triplicate using freshly prepared samples on different days, with a minimum of three replicates per trial. Statistical analyses were conducted using SPSS 27. One-way ANOVA was performed under Duncan’s multiple range test to assess significance at *p* < 0.05. Principal component analysis (PCA) was performed on the data using Origin 2021.

## Figures and Tables

**Table 1:** Essential amino acid composition of GFPs.

Amino Acid (g/100 g)	GFP1		GFP2		GFP3		GFP4		FAO * (g/100 g)
	Content	AAS	Content	AAS	Content	AAS	Content	AAS	
Val	1.90 ± 0.41 a	0.48	1.90 ± 0.15 a	0.47	1.86 ± 0.08 a	0.47	1.62 ± 0.08 a	0.41	4.00
Met	0.39 ± 0.33 a	0.17	0.37 ± 0.26 a	0.16	0.33 ± 0.25 a	0.14	0.35 ± 0.30 a	0.15	2.30
Ile	1.73 ± 0.43 a	0.58	1.76 ± 0.21 a	0.59	1.73 ± 0.10 a	0.58	1.54 ± 0.16 a	0.51	3.00
Leu	3.59 ± 0.46 a	0.59	3.62 ± 0.26 a	0.59	3.11 ± 0.63 a	0.51	3.07 ± 0.14 a	0.50	6.10
Phe	2.67 ± 0.26 a	0.65	2.50 ± 0.15 ab	0.61	2.29 ± 0.08 bc	0.56	2.00 ± 0.07 c	0.49	4.10
Lys	1.75 ± 0.30 a	0.36	1.77 ± 0.16 a	0.37	1.76 ± 0.15 a	0.37	1.52 ± 0.14 a	0.32	4.80
Thr	2.36 ± 0.31 a	0.94	2.39 ± 0.21 a	0.95	2.24 ± 0.10 ab	0.90	1.97 ± 0.08 b	0.79	2.50
His	2.11 ± 0.30 a	1.32	2.02 ± 0.30 a	1.26	1.86 ± 0.19 a	1.16	1.86 ± 0.36 a	1.16	1.60
EAA (%)	16.50 ± 2.41 a		16.32 ± 1.22 a		15.18 ± 1.02 a		13.93 ± 0.53 a		
TAA (%)	39.22 ± 4.41 a		39.75 ± 0.34 a		36.26 ± 0.76 ab		33.64 ± 1.49 b		
EAA/TAA (%)	41.97 ± 1.88 a		41.04 ± 2.78 a		41.86 ± 2.17 a		41.49 ± 2.70 a		

* FAO (2013) was followed [28]. Values in the same line sharing no common letters differ significantly (*p* < 0.05).

**Table 2:** Ingredient formulations of four culture media.

Group	Rice Bran/kg	Wheat Bran/kg	Corn Flour/kg	Cottonseed Hulls/kg	Wood Chips/kg	Fine Soil/kg	Brown Sugar/kg	Gypsum Powder/kg	Total/kg
1	8.32	12.64	27.04	38.40	54.40	16.00	1.60	1.60	160.00
2	11.04	18.40	18.40	38.40	54.40	16.00	1.60	1.60	160.00
3	25.12	9.44	13.60	38.40	54.40	16.00	1.60	1.60	160.00
4	0.00	16.00	16.00	54.40	54.40	16.00	1.60	1.60	160.00

**Table 3:** Herschel–Bulkley parameters for the apparent viscosity of the GFP solutions.

	GFP1	GFP2	GFP3	GFP4
K	0.033 ± 0.001	0.020 ± 0.001	0.015 ± 0.000	0.009 ± 0.001
*n*	0.928 ± 0.008	0.846 ± 0.015	0.995 ± 0.005	0.983 ± 0.009
R^2^	0.9998	0.9992	0.9999	0.9997

## Data Availability

Data will be made available on request.
